# Use of weeds as traditional vegetables in Shurugwi District, Zimbabwe

**DOI:** 10.1186/1746-4269-9-60

**Published:** 2013-08-20

**Authors:** Alfred Maroyi

**Affiliations:** 1 Department of Botany, School of Biological and Environmental Sciences, Faculty of Science and Agriculture, University of Fort Hare, Private Bag X1314, Alice 5700, South Africa

## Abstract

**Background:**

Most agricultural weeds are usually regarded as undesirable and targeted for eradication. However, weeds are useful to human beings as food and traditional medicines. Few studies have been done to document the uses of weeds as traditional vegetables. This study was therefore, done to document indigenous knowledge related to the diversity and use of agricultural weeds as traditional vegetables in Shurugwi District, Zimbabwe, emphasizing their role in food security and livelihoods of the local people.

**Materials and methods:**

Semi-structured interviews, observation and guided field walks with 147 participants were employed between December 2011 and January 2012 to obtain ethnobotanical data on the use of edible weeds as traditional vegetables. Based on ethnobotanical information provided by the participants, botanical specimens were collected, numbered, pressed and dried for identification.

**Results:**

A total of 21 edible weeds belonging to 11 families and 15 genera, mostly from Amaranthaceae (19%), Asteraceae and Tiliaceae (14.3%), Capparaceae, Cucurbitaceae and Solanaceae (9.5% each) were identified. Of the documented edible weeds, 52.4% are indigenous while 47.6% are exotic to Zimbabwe; either semi-cultivated or growing naturally as agricultural weeds in farmlands, fallow land and home gardens. Among the main uses of edible weeds were leafy vegetables (81%), followed by edible fruits (19%), edible corms (9.5%), edible flowers and seeds (4.8% each). The most important edible weeds were *Cleome gynandra*, cited by 93.9% of the participants, *Cucumis metuliferus* (90.5%), *Cucumis anguria* (87.8%), *Corchorus tridens* (50.3%) and *Amaranthus hybridus* (39.5%). All edible weeds were available during rainy and harvest period with *Cleome gynandra*, *Corchorus tridens, Cucumis anguria, Cucumis metuliferus* and *Moringa oleifera* also available during the dry season, enabling households to obtain food outputs in different times of the year. The importance of edible weeds for local livelihoods was ubiquitously perceived, with all participants reporting their contribution towards food security and nutrition.

**Conclusion:**

The present study confirm findings from similar studies conducted elsewhere that rural households engage in harvesting of wild edible vegetables and other non-timber forest products (NTFPs) as a survival strategy. Based on their potential nutritional and medicinal value, edible weeds could contribute in a major way to food security, basic primary health care and balanced diets of rural households and possibly urban households as well.

## Background

A weed is any plant growing where it is not wanted or interfering with the objectives of humans [1]. This means that weeds are undesirable from a human point of view; and grow where other plants are supposed to grow or where no plants should be. This is because weeds normally invade natural vegetation, usually adversely affecting native biodiversity or ecosystem functioning [2,3] or invade agricultural land, impacting on the growth and productivity of cultivated crops. However, weeds are useful to human beings as food, erosion control, medicines, aesthetic value, shelter, supply of organic matter and mineral nutrients to the soil. Consumption of agricultural weeds is a world-wide phenomenon as some of the plants are characterized by high nutritional value and medicinal properties [4]. Agricultural weeds are consumed in several African and Asian countries mainly as vegetables [5-9]. Many rural communities in tropical Africa make use of vegetables to supplement their diet which is based on rainfed cultivation of staples such as cassava, maize, millet, sorghum, and wheat. Research by Ogoye-Ndegwa and Aagaard-Hansen [10] showed that leafy vegetables gathered from the wild form part of the diet of many rural households in Kenya. Similar research in Swaziland showed that a significant proportion of traditional vegetables are collected from agricultural fields, disturbed environments and household gardens [7]. According to Jansen van Rensburg et al. [11], traditional vegetables have been collected from the wild and as ‘weeds’ in agricultural and disturbed spaces for millennia. Traditional vegetables are all plants whose leaves, roots or fruits are acceptable and used as vegetables by rural and urban communities through tradition, custom and habit [12]. Traditional vegetables may not be indigenous to a country, but are usually associated with traditional production systems, local knowledge and have a long history of local selection and usage [13,14].

The food value of agricultural weeds as traditional vegetables in Zimbabwe is in general often ignored and receives little recognition from the government. This might perhaps be due to lack of information about the extent of their use as traditional vegetables and their importance to rural and urban livelihoods. Basic information on diversity and utilization of agricultural weeds as traditional vegetables in Zimbabwe is lacking, despite the growing recognition that agricultural weeds constitute an important component of farmer’s diets around the world [5]. The documentation of how agricultural weeds are utilized by rural communities can serve as an initial step towards further detailed studies on the importance of weeds in agricultural systems. It is in this context that a study was undertaken to document indigenous knowledge related to the diversity and use of agricultural weeds as traditional vegetables in Shurugwi District, Zimbabwe, emphasizing their role in food security and livelihoods of the local people.

## Materials and methods

### Study area

**Figure 1:**
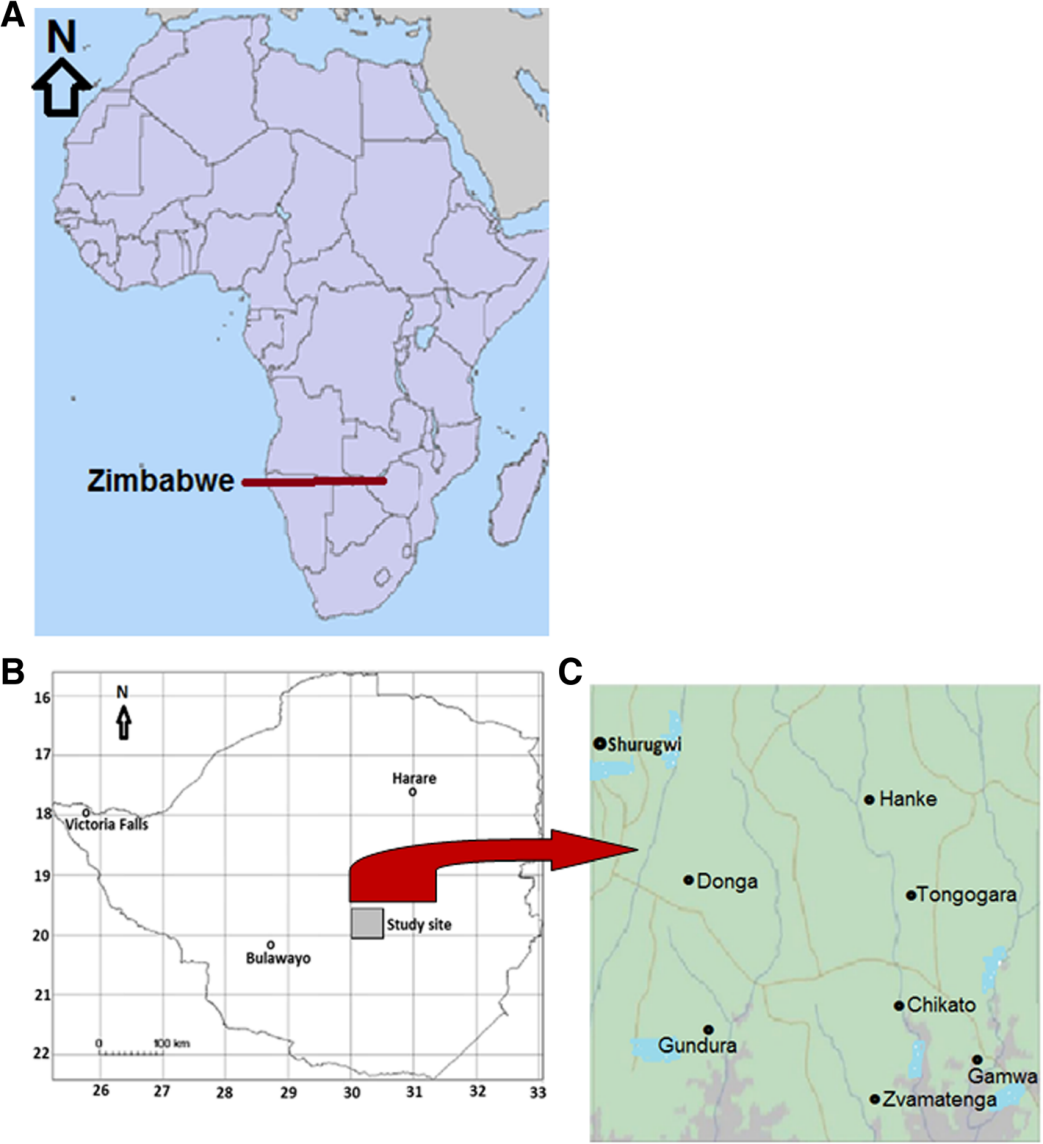
**Geographical location of the study area. A**: Map of Africa illustrating the geographical position of Zimbabwe. **B**: Map of Zimbabwe illustrating the geographical position of Shurugwi District. **C**: Detailed illustration of the study area.

Field studies were carried out in seven communities: Chikato, Donga, Gamwa, Gundura, Hanke, Tongogara and Zvamatenga (Figure 1), all located in Shurugwi District, Midlands Province, Zimbabwe. This investigation is part of a larger study (see Maroyi [15,16]) aimed at documenting ethnobotanical knowledge held by local communities in Shurugwi District, Midlands Province, Zimbabwe. The District Agricultural Extension Officers provided guidance on the final choice of the study areas based on villages who utilized edible weeds. The study area lies between 19°57′S to 20°30′S latitude and 30°00′E to 30°58′E longitude. The study area lies in agro-ecological region 3, a semi-intensive agricultural region characterized by annual rainfall of between 650–800 mm a year [17]. In the hottest month, October, the mean temperature is 31°C, and in the coldest month, July, the mean temperature is 9°C. Severe mid-season dry spells and an unreliable start to the rainy season make the area marginal for maize, tobacco and cotton. Soils are sandy loam largely derived from granitic-gneissic rocks characterized by low agricultural potential due to low fertility, water-holding capacity, low pH and deficiencies in nitrogen, phosphorus, and sulphur [18,19].

The most extensive vegetation type is drier miombo woodland [20], in which *Brachystegia spiciformis* Benth. and *Julbernardia globiflora* (Benth.) Troupin are dominant in terms of basal area, with patches of *Hyparrhenia* spp., *Eragrostis* spp., *Heteropogon* spp. and *Digitaria* spp. grasses. With poverty, low levels of economic activity and the poor quality land that they have available to them, non-farm activities are potentially an important source of income. The poorest families depend on the natural environment for resources such as construction timber, firewood, and fencing materials, as well as their daily food which include insects, wild fruits, vegetables and medicine. Their agricultural practices are essentially subsistence in nature, with land and livestock being the primary household assets. Agricultural mechanization is relatively low, with most farmers using hand tools and oxen for ploughing. Other principal assets include ploughs, ox-drawn carts, wheelbarrows, axes, hoes, and the like. Use of chemical fertilizer and improved seeds is becoming increasingly common, especially among relatively affluent residents. Maize cultivation is the main activity, with other grains such as sorghum (*Sorghum bicolor* (L.) Moench) and millet (*Pennisetum glaucum* (L.) R. Br.) being planted by most households as insurance against poor rains, which in some years are inadequate to produce a good maize crop. Subsistence grain crops are supplemented by household vegetable production e.g., pumpkins *(Cucurbita maxima* Duchesne ex Lam.), covo (*Brassica carinata* A. Braun), rape (*Brassica rapa* L.), cabbage (*Brassica oleracea* L.) and beans (*Phaseolus vulgaris* L.).

### Data collection

Ethnobotanical information on utilization of edible weeds was gathered through semi-structured interviews with residents of Chikato, Donga, Gamwa, Gundura, Hanke, Tongogara and Zvamatenga (Figure 1), all located in Shurugwi District, Midlands Province, Zimbabwe. Prior to any contact with participants, the study and its objectives were introduced to the local traditional leaders. Once the traditional leaders granted permission to proceed, individuals were approached for participation. The households were marked on the map and, beginning with a random number between 1 and 10, every 8^th^ household was selected. Only one member of each household was interviewed. Twenty one individuals from each of the seven communities were interviewed between December 2011 and January 2012. Verbal informal consent was obtained from each individual who participated in the study and the researcher adhered to the ethical guidelines of the International Society of Ethnobiology (http://www.ethnobiology.net). The interviews were conducted in Shona language since the author is a native speaker of the language. The aim and purpose of the investigation was explained to all participants. Interviews were conducted individually whenever possible in an attempt to avoid any direct influences from third parties and to ensure that the data supplied by the participants were as direct and reliable as possible [21]. The meaning of the terms “agricultural weed” and/or “weed” as defined by Drummond [22] were explained to the participants. As in previous studies [15,16], participatory rural appraisal (PRA) methods were used [23] to systematically collect data and information as follows:

1. socio-demographic characteristics of participants;

2. names of the edible weeds collected;

3. methods of preparation of the edible weeds;

4. period of availability of edible weeds;

5. impacts edible weeds have on food security and poverty and;

6. other benefits derived from the collection of edible weeds.

### Plant collection and identification

Field trips were made to the sites where the participants collected the edible weeds. Voucher specimens of plants identified as edible weeds were collected during the field trips when encountered for the first time and again when they were flowering or fruiting, for easy identification. The voucher specimens were processed using standard taxonomic procedures [24,25]. Each herbarium specimen included important parts such as leaves, stems, flowers and fruits whenever available. For small herbaceous plants, the whole plants were collected. These specimens were deposited for future reference at the National Herbarium and Botanic Gardens, Harare, Zimbabwe (SRGH).

### Data analysis

The majority of the data collected in this study were qualitative and descriptive in nature, therefore, they were explained directly. Interview and discussion data obtained from the participants were coded and sorted into themes. Any inconsistencies and unique statements obtained from the participants were noted and given particular attention. Data associated with edible weeds were stored in Microsoft Excel 2007. These data were presented using percentages, frequency, ranking and bar charts.

## Results and discussion

### Socio-economic characteristics of the participants

Table 1 shows the demographic characteristics of the participants. Of the one hundred and forty seven participants, 68.7% were female and 31.3% were male. Their ages ranged from 16 to 87 years, with 52 years as the median. Most (78.9%) of the participants were heading households. The majority of participants were married (69.4%), 17% widowed, 8.8% single and 4.8% divorced (Table 1). The majority of households (64.6%) comprised between 5 and 8 family members, while 22.4% comprised 1 to 4 family members and 12.9% had more than 9 family members (Table 1). The majority (66%) of the participants were educated up to primary level, while 19.7% had attained secondary education, 12.2% were illiterate and 2% had attained tertiary education. More than three quarters of the participants (85%) were unemployed, surviving on less than $100 a month (Table 1). A very small proportion of the participants had constant income as civil servants (2%) and pensioners (4.1%) and their income was more than $150 a month (Table 1).

**Table 1:** Socio-economic characteristics of the study sample, N = 147

**Socio-economic variables**		**Number**	**%**
**Gender**	Female	101	68.7
	Male	46	31.3
**Age (years)**	16-25	9	6.1
	26-35	16	10.9
	36-45	33	22.4
	46-55	47	32.0
	56-65	27	18.4
	66-75	8	5.4
	76-85	6	4.1
	>86	1	0.7
**Relationship to household head**	Head of household	116	78.9
	Spouse	22	15.0
	Children	9	6.1
**Marital status**	Single	13	8.8
	Married	102	69.4
	Divorced	7	4.8
	Widowed	25	17.0
**Household size**	1-2	9	6.1
	3-4	24	16.3
	5-6	51	34.7
	7-8	44	29.9
	>9	19	12.9
**Highest level of education**	No education	18	12.2
	Primary	97	66.0
	Secondary	29	19.7
	Tertiary	3	2.0
**Occupation**	Unemployed	125	85.0
	Civil servant	3	2.0
	Pensioner	6	4.1
	Other	13	8.8
**Combined monthly income**	Less than $50	74	50.3
	$50-$99	58	39.5
	$100-$149	3	2.0
	$150-$199	4	2.7
	More than $200	8	5.4

### Diversity of edible weeds

A total of 21 edible weeds belonging to 11 families and 15 genera were identified in Shurugwi District, Zimbabwe (Table 2). These findings compare favourably with 19 edible weeds recorded in Hondey Valley, eastern Zimbabwe [26]. A large number of edible weeds in the current study (16, 76.2%) are from 6 families (Table 3). Plant families with the highest number of edible weeds were: Amaranthaceae (4 species), Asteraceae and Tiliaceae (3 species each), Capparaceae, Cucurbitaceae and Solanaceae (2 species each). The genera with the highest number of edible weeds were *Amaranthus* and *Corchorus* with three species each, *Cleome* and *Cucumis* with two species each. Family Asteraceae is among the largest plant families worldwide and is known to contribute most of the agricultural and environmental weeds [27]. Similarly, previous research by Drummond [22] and Wild [28] showed that a number of species belonging to Asteraceae, Amaranthaceae, Caryophyllaceae, Chenopodiaceae and Solanaceae were among the common weeds of agroecosystems in Zimbabwe. Heywood [29] examined the patterns and extent of invasions by agricultural weeds and concluded that most weeds come from the largest plant families like the Asteraceae and Poaceae.

**Table 2:** Edible agricultural weeds reported by the people of Shurugwi District, Zimbabwe

**Family/species/voucher number**	**Vernacular name**	**Part used**	**Availability**	**No. of citations (%)**
**Amaranthaceae**				
*Amaranthus hybridus* L.; AM543	Mbuya (S); pigweed (E)	Leaves cooked as vegetable	Rainy/harvest season	39.5
*A. spinosus L.; AM123	Mbuya (S); thorny pigweed (E)	Leaves cooked as vegetable	Rainy/harvest season	8.8
*A. thunbergii* Moq.; AM321	Mbuya (S); poor man’s spinach (E)	Leaves cooked as vegetable	Rainy/harvest season	12.2
*Celosia trigyna* L.; AM289	Mundawarara (S); silver spinach (E)	Leaves cooked as vegetable	Rainy/harvest season	16.3
**Asteraceae**				
*Bidens pilosa L.; AM490	Black jack (E); sine (S)	Leaves cooked as vegetable	Rainy/harvest season	6.1
*Galinsoga parviflora Cav.; AM604	Chickweed (E)	Young leaves and shoots cooked as vegetable	Rainy/harvest season	7.5
*Sonchus oleraceus L.; AM111	Rurimirwemombe (S); snow thistle (E);	Leaves cooked as vegetable	Rainy/harvest season	2.7
**Capparaceae**				
*Cleome gynandra* L.; AM308	Nyovhi (S); spider flower (E)	Leaves and young shoots cooked as leafy vegetable. Leaves and shoots sun dried for later consumption	Rainy/harvest/dry season	93.9
*C. monophylla* L.; AM434	Musemwasemwa (S); spindle pod (E);	Leaves cooked as vegetable	Rainy/harvest season	25.9
**Chenopodiaceae**				
*Chenopodium album L.; AM322	Fat hen (E); mubvunzandadya (S)	Leaves cooked as vegetable	Rainy/harvest season	23.8
**Cucurbitaceae**				
*Cucumis anguria L.; AM449	Muchacha (S); wild gherkin (E)	Leaves and young shoots cooked as leafy vegetable. Leaves and young shoots sun dried for later consumption	Rainy/harvest/dry season	87.8
*C. metuliferus* Naudin AM612	Mugaka (S); spiny cucumber (E)	Edible fruit pulp. Ripe fruit stored for later use	Rainy/harvest/dry season	90.5
**Cyperaceae**				
*Cyperus esculentus* L.; AM361	Pfende (S)	Corm (excluding peel) edible	Rainy/harvest season	10.2
**Malvaceae**				
*Hibiscus articulatus* Hochst. ex A. Rich.; AM122	Derere hambakachere (S); wild hibiscus (E)	Leaves cooked as vegetable	Rainy/harvest season	11.6
**Moringaceae**				
*Moringa oleifera Lour.; AM309	Drumstick tree (E); moringa (S)	Leaves, flowers, fruits and seeds cooked as vegetable	Rainy/harvest/dry season	8.2
**Oxalidaceae**				
*Oxalis latifolia Kunth; AM328	Musauti (S)	Corm (excluding peel) edible	Rainy/harvest season	9.5
**Solanaceae**				
*Physalis angulata L.; AM365	Muguzubheri	Fruit eaten raw	Rainy/harvest season	10.9
*Solanum nigrum L.; AM381	Black nightshade (E); Musungusungu (S)	Leaves, young shoots cooked as vegetable, ripe fruit edible	Rainy/harvest season	13.6
**Tiliaceae**				
*Corchorus asplenifolius* Burch.; AM392	Derere (S); Jute (E)	Leaves and young shoots cooked as vegetable	Rainy/harvest season	6.8
*C. olitorius* L.; AM118	Derere (S); Jute (E)	Leaves and young shoots cooked as vegetable	Rainy/harvest season	4.8
*C. tridens* L.; AM528	Derere (S); Wild jute (E)	Leaves and young shoots cooked as vegetable. Leaves and young shoots sun dried for later consumption	Rainy/harvest/dry season	50.3

*Abbreviations*: *E* = English; *S* = Shona.

Species marked with asterisk (*) are exotic.

**Table 3:** Families with the largest number (more than 2 species) of edible weeds in Shurugwi District, Zimbabwe

**Family**	**Number of edible species**	**%**
Amaranthaceae	4	19.0
Asteraceae	3	14.3
Tiliaceae	3	14.3
Capparaceae	2	9.5
Cucurbitaceae	2	9.5
Solanaceae	2	9.5

Of the documented edible weeds, 52.4% are indigenous while 47.6% are exotic to Zimbabwe. With the exception of *Cucumis metuliferus* and *Moringa oleifera* which are semi-cultivated, the rest are categorized as agricultural weeds [22,28,30]. These edible weedy plant species grow naturally in farmlands, abandoned gardens, homesteads and many other ecological areas where they usually occur as weeds and can exist independently of direct human action. They may be harvested from the wild or from fallow and cultivated fields, or they may be cultivated. Similarly, the majority of traditional vegetables in Kenya exist as weeds of agriculture and are procured from the bushland and previously cultivated farmlands where they are communally gathered, and a few grow in kitchen gardens and along the lakeshores [10].

Interviews with participants revealed that the majority of edible weeds mature quickly and were collected during rainy and harvest season (Table 2). Only four species (19%): *Cleome gynandra*, *Cucumis anguria, Cucumis metuliferus* and *Moringa oleifera* were also available during the dry season, enabling households to obtain food in different times of the year (Table 2). These four species are domesticated or tolerated in home gardens. According to participants, *Cleome gynandra*, *Cucumis anguria* and *Cucumis metuliferus* are deliberately spared during digging, weeding and land clearing activities for the benefits or usefulness they provide to households as traditional vegetables. These species were available during the dry season because the leaves of *Cleome gynandra* and *Cucumis anguria* were preserved for latter use by sun drying (Table 2). Ripe fruits of *Cucumis metuliferus* were stored for 1 to 3 months in shade without any treatment. These preservation procedures extended the shelf-life of the edible parts of weedy plants. According to participants, preserved edible weeds formed an important component of the food resources in dry season when they were out of season and during drought periods. Similar results were obtained by Shava et al. [31], who found sun drying to be an important food preservation procedure, allowing rural communities to fill the food gap during periods of scarcity, particularly in the cold and dry winter season. During such periods of food shortage, traditional vegetables previously preserved by drying become very important in household food security. Research by Mnzava [32] showed that preservation of edible leaves is one of the strategies developed to help face times of food shortages.

**Figure 2:**
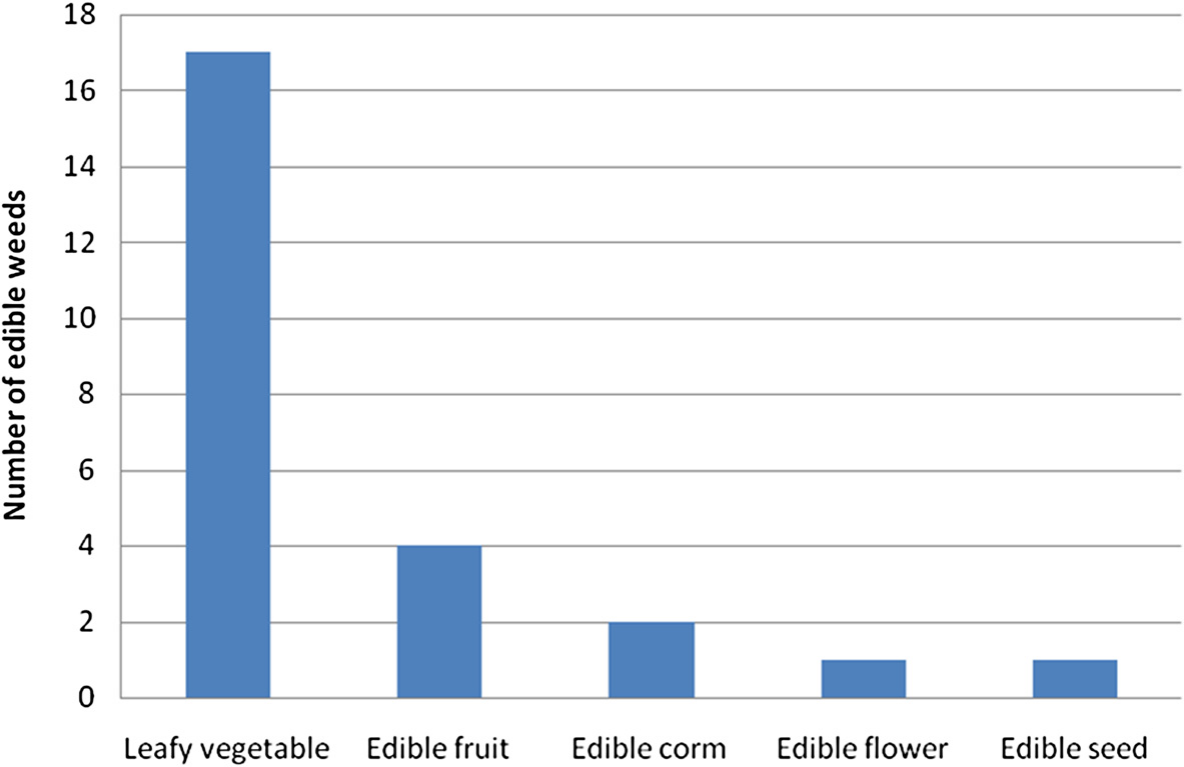
Classification of edible weeds growing in Shurugwi District, Zimbabwe.

Among the main uses of edible weeds were leafy vegetables (81%), followed by edible fruits (19%), edible corms (9.5%), edible flowers and seeds (4.8% each) (Figure 2). The vegetable dishes were prepared mainly as relish which accompanied maize, millet and sorghum porridge. Young leaves and shoots were boiled with salt and fried in cooking oil with other ingredients such as tomatoes and onions. Peanut butter was sometimes used instead of cooking oil. *Corchorus tridens* was cooked with bicarbonate soda or ash to lessen the mucilaginous state of the dishes. When the participants were asked to rate the importance of the species used, the most important edible weeds were *Cleome gynandra*, cited by 93.9% of the participants, *Cucumis metuliferus* (90.5%), *Cucumis anguria* (87.8%), *Corchorus tridens* (50.3%) and *Amaranthus hybridus* (39.5%) (Table 2). However, the most frequently used edible weeds in Hondey Valley, eastern Zimbabwe were *Bidens pilosa*, *Galinsoga parviflora* and *Commelina zambesica*[26]. *Commelina zambesica* was not among edible weeds documented in Shurugwi District, while *Bidens pilosa* and *Galinsoga parviflora* were characterized by low frequency of consumption (Table 2).

These five edible weeds that are regarded as important in Shurugwi District are given special attention and their nutritional value is presented in Table 4. These edible weeds are important sources of macro and micro nutrients which are important for the maintenance of good health and prevention of diseases. The calorific value of 17–308 kcal of the edible weeds (Table 4) compare favourably to 23–84 reported in some South African traditional leafy vegetables [33]; and vegetables in general are known to have low energy values [34]. The protein content of edible weeds (Table 4) is also higher than the protein content of some commercial vegetables [33,35]. Table 4 shows a carbohydrate content range of 2.2-75 g. Only *Cucumis anguria* has a carbohydrate content lower than the 2.65-12.8 g carbohydrate range reported in some South African traditional leafy vegetables [33]. All the five edible weeds have high levels of minerals in comparison to those available in the published literature. For example, only *Cucumis anguria* has calcium content lower than the daily requirement of calcium of 260 mg/day [36]. These substantial amounts of minerals, trace elements and vitamins in the five edible weeds represent potential sources of critical nutrients in the diets of the local community. Previous research by [33], provided evidence that traditional leafy vegetables in South Africa mainly collected from the wild, which do not require formal cultivation could be important contributors to improving the nutritional content of children and other vulnerable groups.

**Table 4:** Nutritional value of the most important edible weeds in Shurugwi District, Zimbabwe

**Species**	**Nutritional value**																	**References**
	**Energy (Kcal)**	**Protein (g)**	**Fat (g)**	**Fibre (g)**	**Carbohydrates (g)**	**Ca (mg)**	**P (mg)**	**K (mg)**	**Na (mg)**	**Mn (mg)**	**Cu (mg)**	**Zn (mg)**	**Fe (mg)**	**Mg (mg)**	**Vit A (mg)**	**Vit B**_**1 **_**(mg)**	**Vit C (mg)**	
*Cleome gynandra*	203	5.1	0.3	1.2	5.2	323	129	20900	-	37.5	8.0	1.0	14.4	119	71.6	0.03	13	[37-40]
*Cucumis metuliferus*	43	4	0.7	2.42	5.55	2974	434	1174	317	4	3	11	20	1022	0.07	0.02	19	[33,41,42]
*Cucumis anguria*	17	1.4	0.3	-	2.2	27	34	-	-	7	-	-	3.6	-	325	0.1	52	[39,43]
*Corchorus tridens*	308	23.6	3.4	8.4	75	1912	308	198	14	4.1	6.2	1.1	6.6	386	73.3	-	78	[37,44]
*Amaranthus hybridus*	53	6	0.5	2.81	6.0	2363	604	4.2	427	24	2	18	21	1317	31.3	2.75	495	[33,45,46]

The remainder of the edible weeds were characterized by low frequency of consumption (Table 2). These included *Amaranthus spinosus* (8.8%)*, Amaranthus thunbergii* (12.2%), *Bidens pilosa* (6.1%)*, Celosia trigyna* (16.3%)*, Chenopodium album* (23.8%), *Cleome monophylla* (25.9%), *Corchorus asplenifolius* (6.8%), *Corchorus olitorius* (4.8%), *Cyperus esculentus* (10.2%), *Galinsoga parviflora* (7.5%)*, Hibiscus articulatus* (11.6%), *Moringa oleifera* (8.2%), *Oxalis latifolia* (9.5%), *Physalis angulata* (10.9%), *Solanum nigrum* (13.6%) and *Sonchus oleraceus* (2.7%). The low frequency of consumption was mainly due to their taste, which was said to be bitter and somewhat discouraging. Similar results were obtained by Łuczaj [47,48] who documented a decline in the use of wild green vegetables in Poland; used mainly in times of food scarcity. The reasons interviewees gave for the continued consumption of these edible weeds in Shurugwi Distict included economics and lack of alternatives. Harvesting of edible weeds in Shurugwi District, Zimbabwe is driven by the fundamental concern to secure food. In this context, the interest in edible weeds is therefore, to mitigate the consequences of insufficient agricultural production. The majority of these edible weeds characterized by low consumption frequency are collectively referred to as “poor man’s food” [31], because they are regarded as inferior and marginalized by the majority of people. Some of these traditional vegetables may be used both as food and medicine. Examples include *Bidens pilosa* and *Moringa oleifera*. Field interviews revealed that some people consume *Bidens pilosa* to ease their “high blood pressure” worries, stomach pains, oral thrush, to boost the immune system and rheumatism.

When preparing a *Cleome gynandra* dish, participants often added *Amaranthus hybridus, Amaranthus spinosus, Amaranthus thunbergii*, *Chenopodium album* and *Cleome monophylla* to increase bulk. Interviews with participants revealed that *Cyperus esculentus, Oxalis latifolia* and *Physalis angulata* were mainly collected and eaten by children. The corms of *Cyperus esculentus* and *Oxalis latifolia* were eaten raw, excluding the peel of the underground parts. Previous research by Campbell [49] and Campbell et al. [50] showed that this opportunistic collection was done by children while undertaking activities such as firewood gathering or water collection.

The importance of edible weeds for local livelihoods was ubiquitously perceived, with all participants reporting their contribution towards food security and nutrition (Table 5). About one fifth (21.1%) reported the importance of edible weeds as traditional medicines and 10.2% reported the contribution of edible weeds towards reduction of poverty levels and food inequalities. A smaller proportion of the participants, 8.2% and 2% reported that edible weeds were sold on local markets to supplement family’s income and exchanged with other goods and services respectively (Table 5). Although perceptions on the actual benefits derived from the collection and management of edible weeds in Shurugwi District were variable among the participants (Table 5), there is no doubt that this category of plant resources is important in meeting household food needs and food security (Table 5).

**Table 5:** Details of the contribution of edible weeds to household livelihoods in Shurugwi District, Zimbabwe

**Uses**	**Response (%), n = 147**
Edible weeds useful for family’s food supply and nutrition	100
Edible weeds useful as traditional medicines	21.1
Edible weeds reduce levels of poverty and inequalities	10.2
Edible weeds sold on local markets supplement family’s income	8.2
Edible weeds exchanged with other goods and services	2.0

Some respondents indicated more than one response.

## Conclusion

This study has revealed that edible weeds play an important role as traditional vegetables in Shurugwi District, Zimbabwe contributing to food and nutritional security of local communities. They are an important part of daily food intake with some preserved for use during the dry season when they are out of season. Edible weeds supplement conventional vegetables. The collection of edible weeds in Shurugwi District has a long history that has been intimately linked to the livelihood needs of the local communities. Some families consume edible weeds not out of choice but due to lack of alternatives. Based on their potential nutritional and medicinal value, edible weeds could contribute in a major way to food security, basic primary health care and balanced diets of rural households and possibly urban households as well. The medicinal value of weeds is well recognized worldwide [51] and there is need therefore, to do more research on the medicinal value of edible weeds and explore the bioactive compounds responsible for the pharmacological effects.

There is a renewed interest on the utilization of weeds in productive ways so that local communities in southern Africa may benefit from an aspect that has been largely ignored for a very long time. Examples include the widespread use of weeds as traditional medicines in South Africa [52,53]. These neglected and underutilized species have begun to attract considerable interest for their multiple underexploited benefits in terms of nutritional value, food security, medicinal and income generation value, availability in large quantities and occupation of marginal environments. The search for novel high quality but inexpensive sources of food has always remained a major concern of all organizations involved in providing food to local communities. In this context, these edible weeds will play a vital role towards food and nutritional security of the nation, as their economic value is beyond dispute. Although the popularity of edible weeds is threatened, knowledge of their uses and preparation is still needed to fully understand the role of indigenous vegetables in both rural and urban communities.

## Competing interest

The authors declared that they have no competing interests.

## Author’s contribution

AM conceptualized the study and wrote the manuscript. The author read and approved the final manuscript.
